# Paradigm shifts in critical care medicine: the progress we have made

**DOI:** 10.1186/cc14728

**Published:** 2015-12-18

**Authors:** Jean-Louis Vincent, Jacques Creteur

**Affiliations:** 1 Department of Intensive Care, Erasme University Hospital, Université libre de Bruxelles, Route de Lennik 808, B-1070 Brussels, Belgium

## Abstract

There have really been no single, major, advances in critical care medicine since the specialty came into existence. There has, however, been a gradual, continuous improvement in the process of care over the years, which has resulted in improved patient outcomes. Here, we will highlight just a few of the paradigm shifts we have seen in processes of critical care, including the move from small, closed units to larger, more open ICUs; from a paternal "dictatorship" to more "democratic" team-work; from intermittent to continuous, invasive to less-invasive monitoring; from "more" interventions to "less" thus reducing iatrogenicity; from consideration of critical illness as a single event to realization that it is just one part of a trajectory; and from "four walls" to "no walls" as we take intensive care outside the physical ICU. These and other paradigm shifts have resulted in improvements in the whole approach to patient management, leading to more holistic, humane care for patients and their families. As critical care medicine continues to develop, further paradigm shifts in processes of care are inevitable and must be embraced if we are to continue to provide the best possible care for all critically ill patients.

## Introduction

ICUs are designed to care for critically ill patients, those requiring more support, attention, and surveillance than is available on the general floor. Because ICUs cater for the needs of the sickest hospital patients, it is not surprising that mortality rates are higher than on the general ward, averaging around 15% across the globe [1]. As medicine progresses, the ICU must continue to accommodate the very sickest patients, those who would not previously have survived long enough to benefit from intensive care. As such, and despite continuing advances in medicine, ICU mortality rates will probably remain fairly static: indeed, if mortality rates decreased, it would rather suggest that ICUs were no longer fulfilling their primary role and were admitting less severely ill patients; they would need to be renamed "surveillance units" or "organ support" units!

It is interesting to note that there have been no major advances in critical care therapeutics over the years despite many attempts. The excitement over the results of activated protein C in sepsis was transient, and it was later withdrawn from the market [2]. Equipment and technology have of course changed considerably. The small mobile respirators of today are unrecognizable compared with the early "tank ventilators" and "iron lungs" [3], and most other forms of equipment have also become smaller and more mobile. Information technology has seen similar expansions into the ICU as in other fields, and is now widely used to speed test requests and result availability and to help reduce errors, such as drug interactions [4]. But perhaps the biggest changes have been in the processes of care within the ICU.

## Paradigm shifts

Paradigms have been defined as "universally recognized scientific achievements that for a time provide model problems and solutions to a community of practitioners" [5]. "Paradigm shifts" occur when situations or "anomalies" occur which no longer fit the expected pattern to such an extent that the original "paradigm" needs to be rethought. Here we discuss some of the major "paradigm shifts" in the processes of intensive care over the past decade or so (Table 1).

**Table 1:** Paradigm shifts in intensive care.

• From small, closed units to larger, more open ICUs
• From a paternal "dictatorship" to more democratic "teamwork"
• Changes in monitoring from intermittent to continuous, invasive to less invasive
• From too much to just enough ...
• Critical illness is not a single phenomenon but part of a patient's disease trajectory
• Expanding beyond the physical ICU structure
• Positive randomized controlled trials are not the only evidence
• Checklists are more helpful than protocols
• Death can be a "good" outcome

### From small, closed units to larger, more open ICUs

The initial ICUs were small, restricted, closed units. Staff (and visitors) had to wear gowns and shoe covers to enter, and even sometimes ring a bell and provide their name through a voice system in order to gain access! Visiting hours were very strict and limited, children were generally not welcome, and in some ICUs family members could only view their relatives through a glass screen! These precautions were primarily "psychological", believing that patients needed to be kept quiet and undisturbed to recover, and also that patients needed to be protected from infections brought in by visitors (and vice versa), a theory based on little scientific evidence--indeed, resistant microorganisms are already present inside the ICU and do not really represent a threat outside, otherwise no-one would want to work in the ICU for fear of taking resistant infections home!

ICUs today are much more accessible and welcoming places for patients, staff, and visitors. Gowns and gloves are rarely seen except when performing sterile interventions. Family visiting hours have been extended in many units, although this not yet a universal finding [6], or even suppressed completely, allowing relatives to visit at any time. Children are also much more welcome.

### From a paternal "dictatorship" to more "democratic" teamwork

In the past, the physician in charge of the ICU too often considered that his (more often a man than a woman) physical presence was essential if patients were to be treated "correctly", and equally that patient management would obviously be suboptimal in his absence. Limited communication among team members meant that quality of care was indeed often better when the chief physician was present than when he was not. The chain of hierarchy meant that other members of the ICU staff were generally there just to follow his orders, and rounds were often punctuated by verbal criticism of more junior doctors.

By contrast, good "teamwork" is now considered the key to achieving high-quality care [7]. All members of the multidisciplinary staff (doctors, nurses, physiotherapists, pharmacists, etc.) have a specific place on the team with a defined role, and all are essential for the effective functioning of the ICU, working together symbiotically. Patient treatment plans are discussed regularly with all members of staff and input from everyone is welcomed and considered. The complexities of critically ill patients and their treatment plans mean that a team approach will always be more efficient, effective, and safer than any one individual trying to cope with the multiple factors involved, much as flying a plane requires not only a pilot but also the rest of the team [8,9].

Although good teamwork is crucial, the presence of a full-time intensivist as team leader is also important and has been associated with improved outcomes [10]. Recognition of intensive care medicine as a specialty in its own right has facilitated development of training programs for specialization of doctors as full-time intensivists. As demand for ICU beds increases, measures need to be taken to ensure there is no shortfall in intensivist numbers in years to come [11]. Nonphysician providers, such as nurse practitioners and physician assistants, are increasingly used, particularly in ICUs in the USA, to carry some of the workload [12], but cannot replace intensivists [13].

### Change in monitoring from intermittent to continuous, invasive to less invasive

Monitoring is a fundamental part of intensive care, but the manner in which patients are monitored has changed fundamentally as technology has developed to meet our changing understanding of basic physiology. Many variables can now be monitored (almost) continuously, rather than intermittently, and continuous traces of multiple variables can be displayed simultaneously on individual patient monitors and fed to central stations within the ICU or even to remote stations in other hospitals. Continuous monitoring ensures that acute changes in a variable are not missed, allows for trends in a particular parameter (e.g., blood lactate or glucose) to be followed, and enables the effects of treatments to be observed more directly. Patient management can thus become less "reactive" and more "proactive", with treatments started early, before a patient deteriorates too much.

Another fundamental change has been the general move from invasive to noninvasive monitoring systems. The development of the pulmonary artery catheter (PAC) was the end of a long search to find a way in which the right heart could be monitored in the clinical arena, and for many years provided the gold-standard means of monitoring hemodynamics, gas exchange, and heart-lung interactions. However, with recent trends towards becoming less invasive, the value of the PAC has been questioned and, with the development of less invasive monitoring systems able to estimate cardiac output and other variables and wider availability of echocardiography, PAC use has decreased worldwide [14,15]. Large randomized controlled trials (RCTs) failed to demonstrate an impact of PAC use on patient outcomes [16,17], but this is not so surprising because the effectiveness of PAC use relies on the accuracy and application of the derived data, which have often been suboptimal, and the availability of effective treatments for the identified condition.

### From too much to less, but still enough

Initial enthusiasm associated with the discovery of an apparently effective new therapy or intervention has sometimes led us to apply the concept that if a small amount works, then more will work better! However, this is not always the case. Over the years, studies have identified various interventions that can increase mortality rates, especially when used in excess. Mechanical ventilation plays a vital role in restoring and maintaining gas exchange in patients with respiratory failure, but can have multiple negative effects, jointly termed "ventilator-induced lung injury" (VILI). The effects of excessive tidal volumes are now well recognized [18]. Similarly, blood transfusions can benefit certain groups of patients by increasing oxygen delivery and can be life-saving in acute hemorrhage, but transfusions can also be associated with transfusion-related acute lung injury (TRALI), transfusion-related cardiac overload (TACO), and transfusion-related immunomodulation (TRIM), and several studies have suggested worse outcomes in patients who receive a transfusion [19]. Other examples include the excessive use of antiarrhythmic agents; overenthusiastic feeding with excessive caloric intakes leading to liver steatosis; and excessive use of sedative agents. Even bed-rest can be seen as a harmful "intervention" when used in excess--the benefits of early mobilization, particularly on longer-term outcomes, have now been reported in many studies [20]. With the realization that sometimes we have been responsible for doing more harm than good, new and established interventions have come under critical review. ICU populations are very heterogeneous and very few interventions can be applied equally to all patients. Rather, patients should be evaluated and treated as individuals with decisions taking into account all of the available clinical, monitoring, and laboratory variables.

### Critical illness is not a single phenomenon but part of the disease trajectory

Until relatively recently, the ICU stay started at the time of ICU admission and stopped at the time of discharge. ICU doctors generally waited until a call was received requesting a transfer to the ICU, and not infrequently blamed their colleagues when the patient was admitted too late after inadequate management on the floor! For the general floor physician, the ICU was often seen as a last resort and referrals only made late in the course of disease. Indeed, critical illness was seen as a "disease" in itself, rather than part of a continuing disease trajectory. As such, signs of deterioration were often not correctly identified or their importance not realized and senior members of staff not alerted in time.

After ICU discharge, patient care was traditionally handed over to the general ward or the general practitioner. But it is well recognized that ICU patients can have major long-term sequellae, not only physical but also psychological [21-23], the so-called "postintensive care syndrome" [24], which can also affect patients' relatives. The paradigm is thus beginning to change and more consideration is being paid to the potential aspects of intensive care that may be associated with worse long-term outcomes. For example, deep sedation was widely used for many years to ensure patient comfort, but when stopped many patients developed delirium, sometimes even necessitating reintroduction of sedative agents! Sedative use can be reduced but this requires a dedicated team approach with good communication and involvement of the patient and families in care processes [25]. A more humane approach to patient care involving greater communication with patients and their families will help reduce some of the long-term psychological burden of critical illness. Patients should not only survive their ICU stay, but survive with minimal sequellae.

### Expanding beyond the physical ICU structure

In the past, ICUs were very much a physical place where patients requiring extra or closer monitoring or specialist therapies, such as mechanical ventilation, were gathered into one place. However, as already seen, critical illness is one part of the disease trajectory and many hospitals now have "medical emergency" or "rapid response" teams that evaluate patients outside the ICU and provide advice regarding early treatment or additional monitoring of deteriorating patients on the ward, to help avert organ damage and potentially reduce the need for ICU admission. The constant shortage of ICU beds has often resulted in admissions of patients too late in the process, sometimes already in multiple organ failure or even after cardiac arrest with brain damage. Yet earlier ICU admission can potentially prevent these complications and shorten ICU stays, thus ultimately resulting in more beds being available! Awareness of the signs and symptoms of deterioration towards critical illness has improved and rapid response teams can help reinforce this. Intensivist input can also be important for conditions that will probably result in ICU admission (e.g., transplantation or cardiac surgery), and also in the management of patients with severe chronic pathologies in an attempt to prevent deterioration requiring ICU admission. These teams can also help instigate discussion regarding goals of care for individual patients, including appropriateness (or not) of ICU admission and end-of-life decision-making [26,27]. The question of intermediate care units is a complex one--although this approach may initially seem to be a solution to limited ICU beds, there is little evidence that it is beneficial in terms of costs or outcomes [28].

Treatment of patients presenting to the emergency room (ER) has also frequently been suboptimal, with long delays in often-crowded ERs. The study by Rivers et al. [29] on early goal-directed therapy for patients presenting to the ER with sepsis was a real eye-opener, demonstrating just how much management could be improved. In our department, we have a special four-bed room (the "shock lab" [30]) where all acutely ill patients are admitted and managed jointly by the ER and the ICU teams. The growing collaboration between the ER (and indeed other hospital wards) and the ICU is an important one and needs to be promoted further to ensure that early management of critically ill patients is assured.

### Positive randomized controlled trials are not the only evidence

Mortality has widely been considered as the best endpoint in clinical trials, because mortality rates are still quite high in many acute diseases. However, mortality is not always the clean, binary endpoint it is often considered to be: it can often be predicted from the time of admission by other factors, including physiological age, degree of severity of the disease, underlying diseases, frailty, or comorbidities. Patients' preferences can further compound the outcome. Most RCTs targeting mortality have given negative results (Table 2), but, importantly, in each arm of the RCT some patients will have benefited from and some patients been harmed by the intervention. For example, in studies on blood transfusion strategies, older patients with underlying coronary artery disease may benefit from more liberal transfusions, whereas younger patients without comorbidities may be harmed by them. Yet in an RCT in general ICU patients, liberal and restrictive arms will include both of these types of patients. Most interventions should take several or multiple factors into account and should thus be individualized, generally on the basis of specific pathophysiological considerations. Our evidence base must include all studies: preclinical and clinical; therapeutic and physiological; interventional and observational.

**Table 2:** Some interventions that have not been shown to be useful in large multicenter trials targeting mortality.

• Tight blood glucose control
• Growth hormone
• Intraaortic balloon counterpulsation
• ScvO_2 _monitoring
• Glutamine administration
• Blood transfusions
• Albumin solutions
• Steroids in septic shock
• Early parenteral nutrition
• NOS inhibitor in septic shock
• Hemoglobin solution in polytrauma
• HES solutions for fluid therapy
• Glutamine supplementation
• Beta-stimulants in ARDS
• Activated protein C in sepsis
• Bicarbonate in metabolic acidosis
• High-frequency ventilation in ARDS
• Antioxidant supplementation
• Craniectomy in severe brain injury
• Talactoferrin in sepsis
• Embolectomy in stroke
• Pulmonary artery catheter

*ARDS *acute respiratory distress syndrome, *HES *hydroxyethyl starch, *NOS *nitric oxide synthase, *ScvO_2 _*central venous oxygen saturation

### Checklists are more helpful than protocols

**Figure 1:**
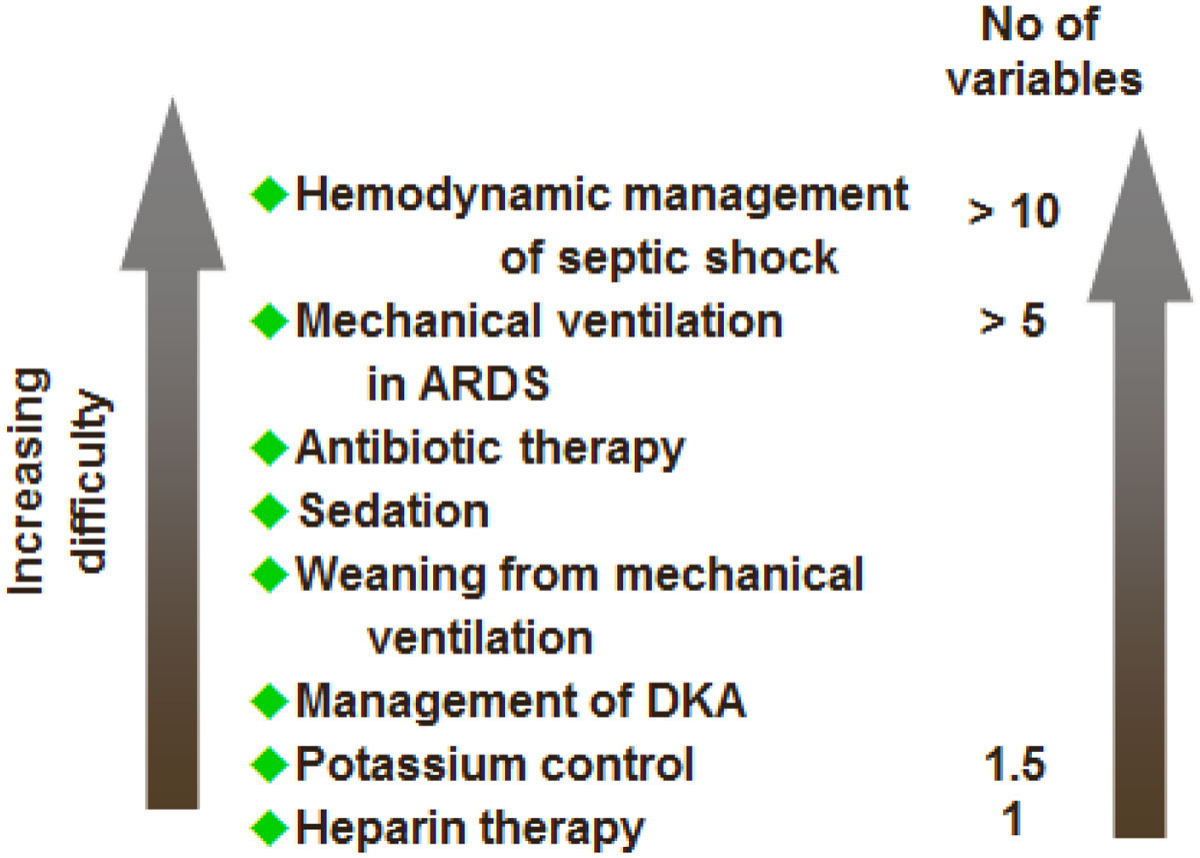
**Some possible areas for protocol-based management in the ICU showing increasing difficulty of protocol, and hence likely reduced usefulness, with increasing number of variables**. *ARDS *acute respiratory distress syndrome; *DKA *diabetic ketoacidosis.

In an attempt to standardize care among staff and across units and hospitals, the concept of protocols was introduced. Paper-based and, more recently, electronic protocols were developed at local, national, and international levels for various aspects of patient management (e.g., sepsis, weaning from mechanical ventilation). However, although protocols can improve the quality of care when it is not very good (especially when fully trained intensivists are not available), they can also decrease the quality of care in units where quality is actually good. For example, a weaning protocol was not shown to be helpful in a teaching environment with generous physician staffing and structured rounds [31]. Protocols are of use for simple therapeutic elements, such as potassium supplementation, but are more difficult to develop and less useable in more complex cases where several variables must be considered simultaneously (Figure 1).

An alternative to the protocol is the checklist. Long checklists may not work very well, because people may tick all of the boxes rapidly without carefully reading the statements, especially when there is pressure on time as is often the case in the ICU situation. A recent study reported that a long safety checklist in the operating room was not associated with a decrease in the incidence of postoperative complications [32]. However, short checklists are easier to use and have been helpful in the aviation industry [33]. Simple checklists, like the FAST-HUG checklist (Table 3) [34], can be helpful to nurses in particular.

**Table 3:** The FAST-HUG checklist [34].

**Feeding**	• Yes/no--how many calories?
**Analgesia**	• Not too much?
**Sedation**	• Can we stop it?
**Thrombosis prophylaxis**	• Unless contraindicated
**Head of the bed elevated**	• Unless contraindicated
**Ulcer prophylaxis**	• Unless contraindicated
**Glucose levels**	• How much insulin?

### Death can be a "good" outcome

Death has long been a taboo subject among patients and their families but also often among doctors and nurses. Moreover, it has often been seen as a sign of failure by the unit, the medical system, and the doctor. As such, the possibility of the patient not surviving the ICU stay has often been pushed aside and prognosis not discussed or, worse, mispresented. But everyone will die one day, and the most important aspect is that we should be allowed to die with dignity and without pain [35]. Survival *per se *is not the ultimate aim of intensive care medicine; rather, the target should be survival with a good quality of life--for patients kept alive with no hope of a meaningful life, death can actually be a good outcome. There is now an increased openness to these issues and people are more willing to confront and discuss difficult end-of-life decisions. Clearly, individual and cultural differences play an important role in such decisions and must be taken into account. With clear communication with families and among the staff, it is sometimes possible to do an ICU test or time-limited trial [36], knowing that if it does not work after a pre-agreed period of time, ongoing life-supporting therapy will be withdrawn [37].

## Conclusion

The processes of care in the ICU have evolved considerably over the years, such that the current ICU would be unrecognizable to our predecessors. There have been no huge advances in therapeutics, but the paradigm shifts discussed have resulted in improvements in the whole approach to patient management, leading to more holistic, humane care for patients and their families. As the need for intensive care continues to increase, key challenges for the future will be to ensure that sufficient ICU beds are available for those who can benefit, with adequate numbers of trained physicians and nurses. There is unlikely to be any single solution to this problem [38], but various factors have been proposed, including: the need for effective admission and discharge criteria; regionalization of ICUs so that the available trained staff are concentrated in fewer larger, high-volume units that may be associated with improved patient outcomes [39] and offer greater staff flexibility; and wider use of telemedicine so that trained doctors from larger ICUs can "virtually" monitor and assess patients on smaller, less well staffed units [40]. Further paradigm changes in processes of care are therefore inevitable if we are to continue to provide the best possible medicine and care for all critically ill patients; part of our role as intensivists is to embrace these positive paradigm shifts.

## Abbreviations

ER, emergency room; PAC, Pulmonary artery catheter; RCT, Randomized controlled trial; TACO, Transfusion-related cardiac overload; TRALI, Transfusion-related acute lung injury; TRIM, Transfusion-related immunomodulation; VILI, Ventilator-induced lung injury.

## Competing interests

The authors declare that they have no competing interests.

## Notes

This article has been published as part of *Critical Care *Volume 19 Supplement 3, 2015: Future of Critical Care Medicine (FCCM) 2014. The full contents of the supplement are available online at http://www.ccforum.com/supplements/19/S3. Publication of this supplement was supported by Fresenius Kabi.
